# Differentiated models of care for postpartum women on antiretroviral therapy in Cape Town, South Africa: a cohort study

**DOI:** 10.7448/IAS.20.5.21636

**Published:** 2017-07-21

**Authors:** Landon Myer, Victoria Iyun, Allison Zerbe, Tamsin K. Phillips, Kirsty Brittain, Elton Mukonda, Joanna Allerton, Cathy D. Kalombo, Andile Nofemela, Elaine J. Abrams

**Affiliations:** ^a^ Centre for Infectious Diseases Epidemiology & Research, School of Public Health & Family Medicine, University of Cape Town, Cape Town, South Africa; ^b^ Division of Epidemiology & Biostatistics, School of Public Health & Family Medicine, University of Cape Town, Cape Town, South Africa; ^c^ ICAP, Mailman School of Public Health, Columbia University, New York, NY, USA; ^d^ Provincial Government of the Western Cape, Cape Town, South Africa; ^e^ College of Physicians & Surgeons, Columbia University, New York, NY, USA

**Keywords:** postnatal care, HIV, antiretroviral therapy, adherence club, maternal and child health, South Africa

## Abstract

**Background**: The numbers of women initiating lifelong antiretroviral therapy (ART) during pregnancy and postpartum is increasing rapidly, presenting a burden on health systems and an urgent need for scalable models of care for this population. In a pilot project, we referred postpartum women who initiated ART during pregnancy to a community-based model of differentiated ART services.

**Methods**: Eligible women (on ART for at least 3 months with viral load (VL)<1000 copies/mL) were offered a choice of two ART models of care: (i) referral to an existing system of community-based ‘adherence clubs’, operated by lay counsellors with medication collection every 2–4 months; or (ii) referral to local primary healthcare clinics (PHC) with services provided by clinicians and medication collection every 1–2 months (local standard of care for postpartum ART). For evaluation, women were followed through 6-months postpartum with VL testing separate from either ART service.

**Results**: Through September 2015, *n* = 129 women were enrolled (median age, 28 years; median time postpartum, 10 days). Overall, 65% (*n* = 84) chose adherence clubs and 35% (*n* = 45) chose PHCs; there were no demographic or clinical predictors of this choice. Location of service delivery was commonly cited as a reason for choice by women selecting either model of care; shorter waiting times, ability to receive ART from lay counsellors and less frequent appointments were motivations for choosing adherence clubs. Among women choosing adherence clubs, 15% never attended the service and another 11% attended the service but were not retained through six months postpartum. Overall, 86% of women (*n* = 111) remained in the evaluation through 6 months postpartum; in this group, there were no differences in VL<1000 copies/mL at six months postpartum between women choosing PHCs (88%) vs. adherence clubs (92%; *p* = 0.483), but women who were not retained in adherence clubs were more likely to have VL≥1000 copies/mL compared to those who remained (*p* = 0.002).

**Discussion**: Adherence clubs may be a valuable model for postpartum women initiating ART in pregnancy, with good short-term outcomes observed during this critical period. To support optimal implementation, further research is needed into patient preferences for models of care, with consideration of integration of maternal and child health services, while ART adherence and retention require ongoing consideration in this population.

## Background

Approaches to the clinical management of HIV infection in pregnant and postpartum women in low- and middle-income countries (LMIC) have undergone a paradigm shift in recent years. From a focus on short-term antiretroviral prophylaxis for the majority of women to prevent mother-to-child HIV transmission (PMTCT), global policies today emphasize universal, lifelong triple-drug antiretroviral therapy (ART) for all pregnant and breastfeeding women [1]. Implementation of this approach has led to rapid increases in the numbers of HIV-infected women receiving ART during pregnancy and the postpartum period [2], placing strain on health systems in many countries [3].

There are significant potential health benefits for maternal and child health (MCH) associated with universal ART initiation by pregnant and breastfeeding women [4]. However, there is mounting evidence that these benefits may be undermined by widespread challenges of non-adherence and/or disengagement from care [5,6]. Multiple studies have suggested that women who initiate ART in pregnancy may be less likely to be retained in care and adherent to treatment, particularly during the postpartum period, compared to other patient populations [7–9]. In turn, identifying approaches to provide effective ART care to women during the postpartum period is a major challenge facing ART programmes globally [10,11].

Outside of pregnancy and the postnatal period, there have been important advances in service delivery models to provide care to increasing numbers of patients on ART in LMIC. This has seen the rise of various models for differentiated ART care, which see stable patients referred to alternative service delivery models which commonly involve less frequent patient contact and are overseen by lay healthcare workers, sometimes taking place outside of established healthcare facilities [12–14]. The “adherence club” is one form of differentiated ART care that has been promulgated in South Africa [15], with evaluations of non-pregnant adults established on ART [16,17]. Differentiated ART care has generated tremendous interest from policymakers as it may be more acceptable to patients and may lead to clinical outcomes that are comparable to those observed under facility-based care, while almost certainly requiring fewer resources to administer [18,19].

While differentiated ART care has tremendous appeal, little is known about its optimal implementation in different health systems and patient populations. In particular, there has been little attention to differentiated ART care for postpartum women, despite their burgeoning numbers and significant concerns around non-adherence and/or disengagement from care in this group. To address this, we carried out a pilot project examining the enrolment of postpartum women into the adherence club system in Cape Town, South Africa.

## Methods

This pilot project and evaluation of postpartum adherence clubs (ClinicalTrials.gov NCT02417675) is nested within the MCH-ART study (NCT01933477), a multicomponent implementation science study examining approaches to ART service delivery for pregnant and postpartum women [20]. The project took place around the Midwife-Obstetric Unit (MOU) of the Gugulethu Community Health Centre (CHC). This facility is located in a low-income, former African township, where the local antenatal HIV prevalence and antenatal care (ANC)/PMTCT coverage are both high [21]. The MOU is operated by nurse-midwives and sees more than 4000 women annually for primary care antenatal and/or obstetric services. Care is provided up to approximately 10 days postpartum, comprised principally of reviewing maternal clinical status as well as infant feeding and wellbeing.

### HIV/ART services

Antenatal ART initiation and follow-up has been integrated into PMTCT services since 2012; from 2013 all HIV-infected women have been eligible for lifelong ART regardless of disease stage or CD4 cell count. The vast majority of women starting ART in pregnancy are initiated onto tenofovir 300 mg, emtracitabine 200 mg and efavirenz 600 mg, taken once daily as a fixed-dose combination. Clinical reviews take place at 1–2 monthly intervals throughout pregnancy, with viral load (VL) testing after 12 weeks on ART.

The local standard of care for ART services during the postpartum period in this setting is referral of mothers at <2 weeks after delivery (depending on the timing of their next scheduled visit to the MOU-ART service) to a network of primary healthcare clinics (PHCs) providing general ART services within the area; their HIV-exposed infants are referred separately to routine “well-baby” services that include routine anthropometry, vaccinations and HIV testing for infants (including HIV PCR at 6–10 weeks and antibody testing at 18 months).

### Adherence clubs

The local model of differentiated ART care in this setting is a community-based adherence club system that has operated since 2012 as part of public sector standard of care [22]. The adherence clubs are facilitated by lay counsellors working in community venues located separately from health facilities. Locally, a network of approximately 105 clubs, each comprised 20–35 patients, meets 2–4 monthly (for an average of 5 visits per year per club) in an community venue located away from the PHC. At each visit, patients are weighed, complete a short symptom review and receive group educational talks on a range of HIV and health-related topics. Prepacked ART is dispensed at each visit and patients may send proxies (treatment buddies) to collect medications on their behalf. Within the club system, a clinical review takes place annually with nurse evaluation and VL testing.

To date, there are more than 3000 patients enrolled into the adherence club system serving the Gugulethu community. Outside of this pilot, these patients are non-pregnant adults who are identified as being eligible for clubs if they have been on ART for at least 6–12 months at a PHC, have a suppressed VL, have no adherence concerns and no comorbidities requiring ongoing clinical management. Individuals who are enrolled in the club system may be referred back to PHC if they miss a club appointment by more than 5 working days or stop taking ART, have an elevated VL (>400 copies/ml), or develop a comorbidity requiring clinical attention [23].

### Evaluation design

Working in partnership with the Gugulethu CHC and provincial health services, we conducted an evaluation of a pilot project allowing women to be referred to adherence clubs immediately postpartum. The evaluation had two discrete components: (i) an initial choice of postpartum ART services and then (ii) a cohort study following all women (regardless of ART service choice) during the postpartum period with regular assessments conducted separately from routine care. In addition to local government approval, this evaluation was approved by the ethics committees of the University of Cape Town and Columbia University.

Eligibility was modelled on the criteria used by the parent MCH-ART study, and included age >18 years, ART initiation in the recent pregnancy, intention to live in Cape Town through 12 months postpartum, and breastfeeding at enrolment. In addition, this evaluation included eligibility for adherence club referral per local practice, most notably documented suppressed VL (based on testing after 12 weeks on ART) and no comorbidity requiring ongoing clinical review.

### Enrolment and choice of postpartum service

Eligible women were identified at routine postpartum care visits taking place at the MOU and were referred to study counsellors for informed consent and an enrolment interview including socio-demographics and medical history. At the end of this interview, women received a standardized counselling script which explained that they could choose the site of their postpartum ART care – (i) referral to the existing system of community-based “adherence clubs”; or (ii) referral to a local PHC. The features of each service were explained briefly, including the location and frequency of visits and dispensing, as well as the care providers involved, and questions were answered. Neither service provided specific care for postpartum women or their children; for women choosing either service, routine infant follow-up including infant HIV testing took place at separate “well-baby” services. Following their decision, a subset of consecutive women completed a short questionnaire on the reasons for their choice of service.

After making their choice, women were referred by the MOU to either the adherence club system or the local PHC. Following local standard of care, referral procedures included counselling on the referral process and the provision of a referral letter, as well as counselling on ART adherence and PMTCT, with provision of 1–2 months ART. Women were then accompanied by a counsellor to make their first appointment for either the next available AC meeting or at the PHC.

### Follow-up

As part of the evaluation, all women were followed in a series of study measurement visits scheduled and conducted separately from either ART service. These visits included assessments of MCH service utilization, ART adherence, as well as VL testing conducted separately from VL monitoring in routine care, and batch tested by the South African National Health Laboratory Services using the Abbott RealTime HIV-1 platform (Abbott Laboratories, Chicago, Illinois, USA). At any study measurement visit where a participant was identified as being non-adherent and/or disengaged from care, counselling was provided on the importance of ART for MCH, with referral back to the selected service, as appropriate.

As part of the evaluation, engagement of women in the adherence clubs was assessed using paper-based registers maintained by lay counsellors at the adherence clubs. We used the definition of non-retention employed by the adherence clubs, based on being more than 5 working days late to a scheduled meeting, to determine when a patient would be removed from the adherence club system and referred back to the local PHC.

### Analysis

Data were analysed using R (Gnu Project). Descriptive statistics included medians with interquartile ranges (IQR), means with standard deviations (SD) and proportions with 95% confidence intervals (CI). The factors associated with (i) initial choice of services and (ii) short-term outcomes in either service were examined with t-, rank sum or Fisher’s exact tests, as appropriate. All statistical tests were two-sided at alpha = 0.05. Mixed effects logistic regression was used to examine the associations between service delivery models and elevated VL after accounting for repeated measures and adjusting for covariation by socio-demographic and clinical characteristics.

## Results

**Figure 1.**
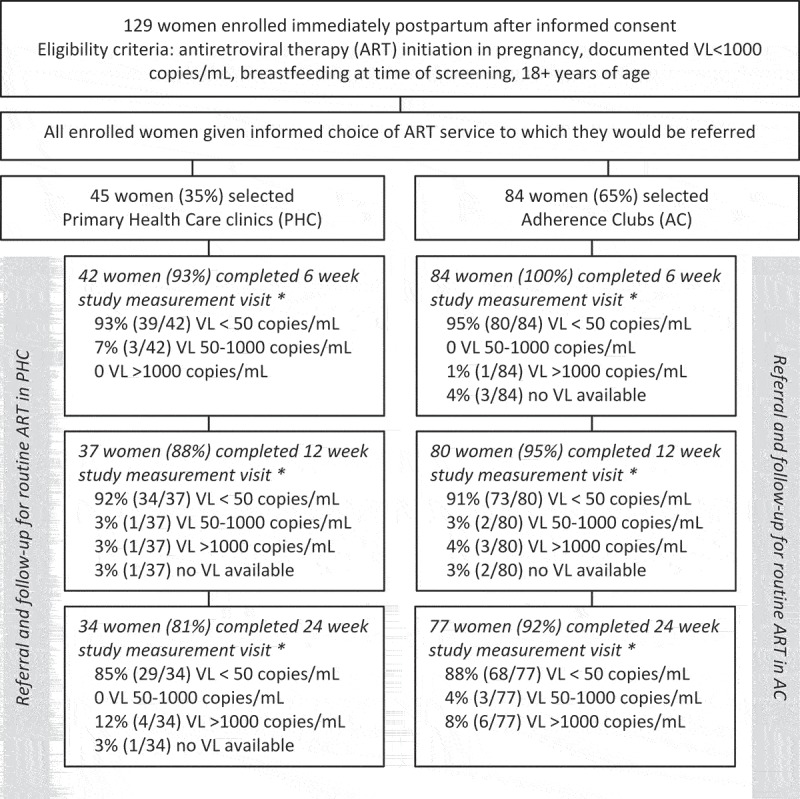
Flow diagram of evaluation procedures. * Study measurement visits conducted by evaluation staff separate from either routine ART service (PHC or AC).

Between February and September 2015, we enrolled 129 postpartum women on ART into the evaluation. Women were enrolled at a median of 10 days post-delivery (IQR, 5–19) with a median of 23 weeks on ART (IQR 18–27). Figure 1 shows the disposition of participants in the evaluation, and Table 1 describes the demographic and clinical characteristics of the sample overall and by choice of ART service. The mean age of women was 28.4 years (SD, 5.2), 20% of women were primiparous, 63% of women were not married or cohabiting, and most women (59%) were newly diagnosed with HIV in the recently ended pregnancy.

**Figure 2.**
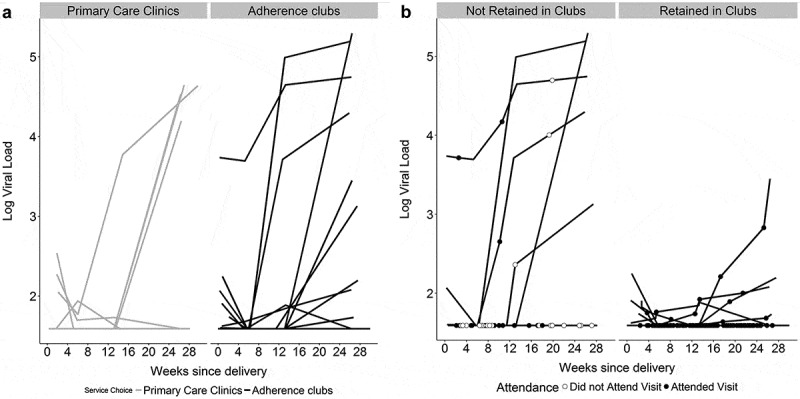
Spaghetti plots of viral load measures conducted at study measurement visits in the cohort (total *n* = 129), from the time enrolment through approximately 6 months postpartum. Panel A shows log_10_ viral loads in women choosing primary care clinics versus adherence clubs. Panel B shows log_10_ viral loads in the cohort of women choosing adherence clubs, separating women who remained engaged in adherence clubs versus those who were not retained through 6 months after deli very. (a) Log10 viral loads in women choosing primary care clinics versus adherence clubs, by weeks from delivery; each line represents a participant. (b) Log10 viral loads among women choosing adherence clubs, separating women who remained engaged in adherence clubs (right-hand panel) versus those who were not retained through 6 months after delivery (left-hand panel); each line represents a participant.

**Table 1.** Demographic and clinical characteristics of postpartum women participating in pilot evaluation, overall and by selected postpartum ART service, in Cape Town, South Africa

		Women selecting		
Variable	All women	*Adherence clubs*	*Primary care clinic*	*p*-Value
Number of participants	129	84 (65)	45 (35)	
Median (IQR) age in years	28 (24–31)	29 (24–31)	28 (24–32)	
<25 years	35 (27)	22 (26)	13 (29)	0.466
25–34 years	79 (61)	50 (60)	29 (64)	
≥35 years	15 (12)	12 (14)	3 (7)	
Home language				
isiXhosa	127 (98)	82 (98)	45 (100)	0.542
Other	2 (2)	2 (2)	0 (0)	
Educational attainment				
Less than secondary	69 (53)	42 (50)	27 (60)	0.278
Completed secondary/any tertiary	60 (47)	42 (50)	18 (40)	
Employment status				
Not employed	84 (66)	51 (61)	33 (73)	0.176
Currently employed	44 (34)	32 (38)	12 (27)	
Relationship status				
Married/cohabiting	48 (37)	32 (38)	16 (36)	0.776
Single	81 (63)	52 (62)	29 (64)	
Parity				
Primiparous	26 (20)	18 (21)	8 (18)	0.622
Multiparous	103 (80)	66 (79)	37 (82)	
Pregnancy intention				
Unintended	88 (68)	59 (70)	29 (64)	0.501
Intended	41 (32)	25 (30)	16 (36)	
Newly diagnosed HIV+ in pregnancy				
Newly diagnosed	76 (59)	48 (57)	28 (62)	0.576
Diagnosed previously	53 (41)	36 (43)	17 (38)	
Any ARV use before pregnancy				
No previous ARV use	90 (70)	58 (69)	32 (71)	
Previous use of ARVs for PMTCT only	27 (21)	17 (20)	10 (22)	
Previous use of ART	12 (9)	9 (11)	3 (7)	0.801
Previous TB diagnosis				
Previously diagnosed	1 (0.8)	0 (0)	1 (2)	0.349
Not previously diagnosed	128 (99.2)	84 (100)	44 (98)	
Median (IQR) weeks on ART	23 (18–27)	23 (19–27)	24 (18–27)	0.927
Median (IQR) days postpartum	10 (5–19)	9 (5–21)	11 (6–15)	0.772
CD4 cell count nearest enrolment (*n *= 124)				
≤350 cells/µL	54 (44)	34 (42)	20 (47)	
>350 cells/µL	70 (56)	47 (58)	23 (53)	0.628
HIV viral load at enrolment				
<50 copies/mL	114 (93)	74 (93)	40 (93)	
50–1000 copies/mL	8 (7)	5 (6)	3 (7)	
>1000 copies/mL	1 (0.8)	1 (1)	0 (0)	1.000
Reported missed ART dose in 30 days prior to enrolment				
No missed doses	110 (85)	71 (85)	39 (87)	
1 or more missed doses	19 (15)	13 (15)	6 (13)	0.743
Intimate partner violence (IPV)				
No IPV in preceding 12 months	113 (88)	76 (90)	37 (82)	0.175
Any IPV in preceding 12 months	16 (12)	8 (10)	8 (18)	

All cells are *N* (%) unless specified otherwise.

### Choice of postpartum services

When offered the choice of postpartum ART services, 65% of women chose to attend the adherence clubs and 35% of women chose to attend the local primary care ART service. There were no clear demographic or clinical predictors of this choice (Table 1). Although women choosing adherence clubs were slightly more likely to be employed and were slightly better educated, neither of these associations achieved statistical significance. Table 2 shows the reasons for the choice of postpartum ART service, among 58 women with data available. The defining features of the respective services – in terms of visit frequency, type of healthcare providers, and the availability of peer support – were frequently cited by individuals choosing either service. For example, 66% of women choosing adherence clubs cited the preference to receive care outside of the clinic, while 36% of women choosing PHCs stated that they did not wish to receive community-based care.

**Table 2.** Women’s reasons for preferring adherence clubs or primary care clinics for postpartum ART services, in a subset of women completing questions on the reasons for their choice.

Adherence clubs		Primary care clinics	
Reason for choice	*n* (%)	Reason for choice	*n* (%)
*Number of participants who gave reasons for service choice*	*44*	*Number of participants who gave reasons for service choice*	*14*
I live closer to the club	26 (59)	I live closer to the clinic	14 (100)
The club location is further than the clinic, from where I live	9 (20)	The clinic is further than the clubs venue, from where I live	0 (0)
I prefer to receive care outside of the health facility	29 (66)	I prefer not to receive care from the community	5 (36)
I want to get peer support	23 (52)	I don’t want peer support	6 (43)
You have a few appointments in a year	32 (73)	I want regular check-ups	9 (64)
Visits are short	41 (93)	I don’t have enough information on clubs	4 (29)
I’m happy to receive my ART care from a counsellor	35 (80)	I want to see a doctor/nurse frequently	11 (79)
I don’t need to see a doctor/nurse frequently	17 (39)	I don’t want to receive care from a counsellor	3 (21)
The club is closer to where my baby receives care	18 (41)	The clinic is closer to where my baby receives care	10 (71)
A friend/family member goes to the club	2 (5)	A friend/family member goes to the clinic	4 (29)

Among women choosing either service, geographic location was frequently noted as a reason for their choice – for 100% and 59% of women choosing clinics and clubs, respectively. However, a further 20% of women choosing clubs noted that a desirable feature was that these were located further from their home than the PHC. In addition, substantial proportions of women in each group stated that their reason for choosing either service was the proximity to local “well-baby” clinical services for their infants.

### Viral load during the postpartum period by choice of service

After enrolment and the initial choice of and referral to an ART service, 98%, 91% and 85% of women were followed at study measurement visits, separate from either ART service, held at 6, 12 and 24 weeks postpartum, respectively. There were no significant differences between women who did and did not complete the 6 month study measurement visit (Supplemental Table 1). The results of VL testing at these study measurement visits are shown in Table 3 and Figure 2(a), overall and by chosen referral service. The proportions of women with VL>50 and >1000 copies/mL both increased over time. For example by 6 months postpartum, VL>1000 copies/mL was observed in 8% versus 12% of women choosing clubs versus PHCs, respectively (*p* = 0.483). In multivariable models, the absence of an association between chosen ART service and elevated VL through 6 months postpartum persisted (Supplemental Table 2).

**Table 3.** Results of viral load testing conducted at study measurement visits, separate from chosen ART service, by postpartum ART service

	Total	Adherence clubs	Primary care clinic	*p*-Value
**HIV viral load at 6 weeks postpartum**				
<50 copies/mL	119 (97)	80 (99)	39 (93)	
50–1000 copies/mL	3 (2)	0 (0)	3 (7)	
>1000 copies/mL	1 (0.8)	1 (1)	0 (0)	0.038
**HIV viral load at 3 months postpartum**				
<50 copies/mL	107 (94)	73 (94)	34 (94)	
50–1000 copies/mL	3 (3)	2 (3)	1 (3)	
>1000 copies/mL	4 (4)	3 (4)	1 (3)	1.000
**HIV viral load at 6 months postpartum**				
<50 copies/mL	97 (88)	68 (88)	29 (88)	
50–1000 copies/mL	3 (3)	3 (4)	0 (0)	
>1000 copies/mL	10 (9)	6 (8)	4 (12)	0.504

All cells are *N* (%).

### Retention in adherence clubs

Of the 84 women who chose to attend adherence clubs immediately postpartum, 15% (*n* = 13) were found on medical record review to have never attended any adherence club meeting, and an additional 11% (*n* = 9) were not retained in the club system following their initial meeting. The demographic and clinical characteristics of these 22 women – comprising 26% of all women who chose clubs – were largely similar to women who remained in the club system, although women who remained in the club system were more likely to be employed than women who did not (*p* = 0.039). Women who were not retained in the adherence club system were more likely to have elevated VL at 6 months postpartum (28%) compared to women who were retained (2%; *p* = 0.002; Figure 2(b)).

## Discussion

This evaluation provides insights into the implementation of differentiated ART care for postpartum women who initiated therapy during pregnancy, a patient population who may be at particular risk of non-adherence and disengagement from care. The key findings are that patient preferences for models of care varied, but that short-term VL outcomes among women choosing adherence clubs appeared broadly comparable to those among women choosing primary healthcare clinics.

There are few documented experiences of patients choosing from different models of ART care, including differentiated models of care. Interestingly, there were no factors strongly associated with the choice of postpartum services but location-related preferences appeared prominent – with most women citing the location of either service as a reason for their choice. Given that participants in this evaluation had not experienced either the adherence clubs or the ART services available at primary care clinics, it may be understandable that location emerges as an important basic consideration. While geography is a well-known determinant of health services uptake, acceptability and outcomes [24], it is possible that in other care contexts the most common reasons for preferring or avoiding differentiated models of care may vary.

We found similar VL outcomes over the first six months postpartum when comparing women attending adherence clubs to those choosing primary care clinics. Importantly VL was measured separately from routine ART care, allowing an independent evaluation of ART effectiveness under either model. However, the comparison of VL outcomes must be framed in the context of patient self-selection into their preferred service, and as such, the outcomes observed may represent a “best case” scenario under both the adherence club system and primary healthcare clinics. And while the follow-up period here – through 6 months postpartum – captures the critical window of the immediate postnatal period, data on longer-term virologic outcomes are required.

In this evaluation, approximately one-quarter of women who chose the adherence club system for postpartum care were not retained in the adherence clubs through 6 months postpartum. This finding parallels the well-known challenges in providing care to this population, but also highlights a key consideration in transferring patients to differentiated ART care. Specifically, we found that 15% of women who chose the adherence club system and who received an appropriate referral never attended an adherence club. This proportion is surprisingly high – particularly given that women were allowed to choose their postpartum service – and loss to follow-up immediately after this initial transfer has not been documented previously in examinations of differentiated models of care. Instead, previous published evaluations typically begin with patients making their first visit to the differentiated service [16,17]. This evaluation suggests that such approaches are likely to overestimate retention of patients in care and should be avoided if possible. More generally, while challenges in linking ART-eligible patients to ART care are well known around treatment initiation, the challenges in transferring patients between ART services postpartum has received less attention [25,26] and we expect this issue to emerge as an important consideration with increasing numbers of patients referred to differentiated ART care.

### Emerging questions for differentiated ART care

It is important to note that in this pilot project, women who initiated ART in pregnancy were referred immediately postpartum to an existing system of adherence clubs. These features raise at least two important issues that speak to broader concerns in the design and delivery of differentiated ART care.

#### How soon after ART initiation can patients be referred to differentiated models of care?

In enrolling women who initiated ART during pregnancy immediately postpartum, the median duration of ART use before referral to differentiated care was relatively short; to date, other models typically involve patients established on ART for substantially longer periods [16,17]. The question of how soon ART patients may be referred to a differentiated model of care is not well understood but will be a major question emerging in settings that are implementing universal ART eligibility and encountering sizable numbers of new ART patients. While this evaluation does not provide direct evidence on whether duration of ART may influence outcomes under differentiated care, it does suggest that early referral of patients on ART to such services may be feasible in this context. Pregnancy and the postpartum may be unique in this regards, however, since the end of pregnancy marks a period of change in many health service settings, with the end of intensive woman-focused antenatal care and the start of infant-focused MCH services. More broadly, we posit that at least some patients may be referred to differentiated ART care very rapidly after initiation while others would benefit from a more substantial foundation of experiencing traditional facility-based services for an extended period. Current approaches to identifying patients suitable for differentiated care, such as early VL suppression on routine monitoring, may only distinguish a fraction of such patients; understanding the characteristics of patients who fare well in differentiated care (and those who may not) is an important future research direction.

#### Should models of differentiated care be customized to special populations?

In this pilot project, the adherence club system to which women were referred was designed for the general population of adults on ART and did not include any adaptations to cater to specific maternal and/or child healthcare needs. This approach was based on consultation with the local health services who explicitly wished to avoid the potential programmatic and operational complexity associated with creating “special” clubs for postpartum women separate from the established public sector model of differentiated ART care. However sending postpartum women into the mix of general adult ART patients may not be ideal in all instances, and there are opportunities to provide MCH services within adherence clubs for postpartum women [27]. Such MCH-focused adherence clubs might include family planning for mothers on ART, infant feeding counselling, and HIV PCR testing as well as vaccinations and anthropometry for infants.

This possibility of special MCH-focused adherence clubs raises significant and broader questions related to the fundamental nature of differentiated ART care: if a differentiated model seeks to address the comprehensive set of primary healthcare needs facing different types of patients on ART, that model has the potential to become hypertrophied to the point of approximating a primary care health clinic. Such differentiated models of care may risk losing the focused efficiency which makes the approach appealing to many patients, providers and policymakers. This tension is not unique to postpartum women, and is likely to permeate the discussions around differentiated ART care for other special populations where the population in question has specific, significant healthcare requirements beyond basic ART provision. How this tension around the customization of differentiated models of ART care is best negotiated is highly context specific, based in part on local health systems as well as resource availability. Patient outcomes associated with greater versus lesser degrees of customization will be an important focus for research programmes in the future.

### Strengths & limitations

These experiences are subject to a number of limitations. The study took place in one part of urban South Africa and findings should be generalized with caution, although the adherence club system evaluated here is well established within local public health services and did not benefit from donor or NGO support. While this is one of the first evaluations of postpartum women attending adherence clubs, the sample size is small and in turn our power to detect quantitative associations are limited. And while the evaluation is strengthened by the use of VL measures carried out independently from routine care, these outcomes were assessed over a relatively short time period, and there is a clear and urgent need for longer-term follow-up as well as inclusion of more diverse outcomes for both mothers and their infants.

## Conclusions

In summary, findings from this pilot project suggest that the enrolment of postpartum women

Into adherence clubs may be a feasible model for women initiating ART in pregnancy, with short-term outcomes observed during this critical period that are comparable between adherence clubs and local primary care clinics. To support optimal implementation, further research is needed to understand the role of patient preferences for various models of care, as well as the issues involved in combining differentiated care alongside routine MCH services, while ART adherence and retention during the postpartum period require ongoing attention.

## Notes

To access the supplementary material to this article please see Supplementary Files under Article Tools online.
